# West Syndrome: Response to Valproate

**DOI:** 10.3389/fneur.2012.00166

**Published:** 2012-11-23

**Authors:** Surabhi Chandra, Anupama Bhave, Roli Bhargava, Chandrakanta Kumar, Rashmi Kumar

**Affiliations:** ^1^Department of Pediatrics, CSM Medical UniversityLucknow, India

**Keywords:** West syndrome, infantile spasms, treatment, predictors

## Abstract

Management of West syndrome is unsatisfactory. In our clinic we observed that a significant proportion of patients respond to usual dose of valproate. **Objective:** To prospectively assess the efficacy of valproate in controlling infantile spasms in West syndrome. **Methods:** Consecutive patients presenting with West syndrome to the Pediatric Neurology Clinic or general outpatient department (OPD) were enrolled for study. Those who were not on any treatment were given valproate in a dose of 30 mg/kg/day while awaiting investigations. Patients were followed up every 2 weeks. Predefined criteria for definition of West syndrome and response were used. Those showing partial/poor response or relapse on valproate were given hormonal therapy. **Results:** One hundred children with West syndrome were enrolled. Ninety one children were started on valproate. Of these 36 (39.5%) showed a good response, but seven later relapsed while on same dose of valproate and three were lost to follow up. Later age at onset and typical hypsarrhythmia on EEG were associated with good sustained response to valproate while a history of delayed cry at birth was associated with partial or poor response. Sixty two patients who responded poorly to or relapsed on valproate were put on hormonal treatment in addition. Of these 36 (58.1%) had a good response but 11 later relapsed after stopping treatment and two were lost to follow up. **Conclusion:** Valproate may have a role in treatment of West syndrome in a selected group of patients.

West syndrome is an age-dependent epileptic encephalopathy characterized by the clinico-electrical triad of infantile epileptic spasms, arrest, or regression of psychomotor development and hypsarrhythmia, although the latter element may be missing (Lux and Osborne, 2004). Variants of this classical triad comprise variations of age of onset, ranging from the first month to 4 years; spasms that may be single, asymmetrical, or combined with focal seizures; asymmetrical, synchronous, or fragmented hypsarrhythmia; and psychomotor function which may be delayed, deteriorated, or normal (Lux and Osborne, 2004).

West syndrome is the most common infantile epileptic syndrome seen in our Pediatric Neurology Clinic. In a study from the All India Institute of Medical Sciences, New Delhi, it was the commonest epileptic encephalopathy seen in the pediatric neurology clinic (Kalra et al., 2002). The same is the experience at our pediatric neurology clinic at the King George Medical University, Lucknow.

Although West syndrome is a common epileptic syndrome, its treatment is so far unsatisfactory. ACTH or oral steroids are two commonly used drugs controlling epileptic spasms in about two-thirds of patients within days of initiation, while vigabatrin is especially useful in tuberous sclerosis associated West syndrome (MacKay et al., 2004). However, a significant number of patients relapse after discontinuation and no studies till date show conclusively that any one treatment significantly improves the long-term intellectual outcome of these infants (Hancock et al., 2009). There are some studies on use of valproate in West syndrome (Bachman, 1982; Dyken et al., 1985; Seimes et al., 1988; Pratz et al., 1991; Ohtsuka et al., 1992; Sharma, 2012). In our own Pediatric Neurology Clinic we found that in a significant proportion of patients, the spasms respond completely to usual dose of valproate while awaiting investigations. We therefore embarked on a systematic study on treatment of West syndrome to prospectively assess the response to usual dose valproate and study factors predicting such a response.

## Materials and Methods

### Setting

The study was conducted in the pediatric outpatient department (OPD) and Pediatric Neurology Clinic of the King George Medical University Hospital, Lucknow. Lucknow is the capital of the state of Uttar Pradesh – India’s most populous state and also one of the poorest. The King George Medical University Hospital is a state run teaching hospital which caters to poor or seriously sick patients from Lucknow city and the surrounding districts.

Children aged 1 month to 5 years attending either the general pediatric OPD or the Pediatric Neurology Clinic were enrolled for the study if they had infantile spasms and psychomotor retardation or regression with or without hypsarrhythmia on EEG. Acutely sick children, and those whose parents did not consent for the study were excluded. Patients already receiving antiepileptic drugs were enrolled but excluded from the comparison of factors predicting response to treatment. Socioeconomic status was judged by the Kuppuswamy scale (Phatak, 1997). A history of perinatal asphyxia, low birth weight, and prematurity was taken from medical records or if these were unavailable, by simply questioning the mother and other family members.

Enrolled patients were worked up according to a standardized protocol. Details of history, examination, investigations, and follow up were entered in predesigned data collection forms. A 16 channel EEG and neuroimaging (magnetic resonance imaging of brain – 1.5 Tesla machine or cranial CT scan – as afforded by the family) was obtained. Tests for developmental quotient (developmental Assessment of Indian Infants – DASII; Malin, 1971) and social quotient (Malin’s adaptation of Vineland Social Maturity Scale; Matsuo et al., 2001) were administered by a trained child psychologist initially and at the end of 6 months.

At the first visit, previously untreated patients were started on valproate syrup in a dose of 30 mg/kg/day in three divided doses while awaiting investigations.

### Follow up

The patient was asked to keep a seizure dairy and recalled every 2 weeks. On each visit the number of series/day and number of spasms per series was recorded. Note was made of occurrence of any other types of seizures or relapse of infantile spasms.

Spasm Outcome was assessed as follows:

1. No reduction in spasms was considered as no response.
2. If spasms decreased by less than 50% after initiation of therapy, it was considered as a poor response.
3. If spasms decreased by more than 50% but less than 80% after initiation of therapy, it was considered as a partial response.
4. If spasms decreased by more than 80%, it was considered as good response.
5. Complete cessation of spasms was classified as complete response.

Response categories 1, 2, and 3 and those who relapsed after initial good response were considered as partial or poor responders. In these patients, valproate was continued in same doses and hormonal therapy was added. Response categories 4 and 5 were considered good responders if they did not relapse and were continued on valproate alone. Hormonal treatment was in the form of daily injection ACTH 40 units intramuscularly or oral prednisolone 2 mg/kg/day in 2–3 divided doses for 2 weeks. If spasms were controlled within this period the ACTH or prednisolone was tapered over the next 2 weeks. In case of partial or poor response these medications were continued for a total of 4 weeks and then tapered.

The study had the approval of the Ethics Committee of King George Medical University, Lucknow. Written informed consent was taken from parents/guardians of the patient.

### Analysis

Patient and spasm characteristics were compared between responders and non-responders to arrive at factors associated with good sustained response to valproate. The two sample *t* test was used for comparing means and the Chi-square test was used for comparing proportions.

## Results

A total of 100 patients were enrolled during the study period and the clinical profile and demographic factors were studied in all. The clinical profile and the demographic factors are described in Table 1. The mean age of onset of spasms was 5.77 months and the mean age of presentation of the patients to us was 14.97 months, mean delay in presentation being 9.29 months. Adverse perinatal/neonatal events had occurred in almost three fourth of the patients, while another 15% had a history of adverse post-neonatal events (post-neonatal central nervous system infection in eight and significant head injury in two). Classic hypsarrhythmia was present in 1/3rd of cases only. Sequelae to hypoxic ischemic encephalopathy (HIE) was the most common abnormality detected on neuroimaging.

**Table 1:** **Descriptive data on 100 enrolled patients**.

Factor	No. of patients
Mean age at onset in months (SD)	5.77 (6.78)
Mean age at presentation in months (SD)	14.97 (10.84)
Mean delay in presentation in months (SD)	9.29 (8.85)
Male sex	77
Urban residence	46
Lower socio economic class	38
Upper socio economic class	4
Adverse perinatal/neonatal events	72
Assisted vaginal delivery	3
LSCS delivery	16
Preterm gestation	9
Low birth weight	27
Delayed timing of cry at birth	65
History of neonatal illness	52
Adverse post-neonatal events	15
**TYPE OF SPASMS**	
Flexor spasms	85
Extensor spasms	9
Mixed spasms	6
Spasm occurrence in series	52
Associated features with spasms	57
Spasms mostly on awakening	81
**ASSOCIATED FEATURES**	
Developmental delay at onset of seizures	88
Any dysmorphic feature	23
Microcephaly	55
Abnormal motor examination	73
Other seizure types at presentation	20
**NEUROIMAGING (DONE IN 88 PATIENTS)**	
Normal	1/88 (1.1)
Atrophy	26/88 (29.5)
Encephalomalacia/sequel to HIE	27/88 (30.6)
Infarct/focal atrophy/scar	17/88 (19.3)
Malformation of brain	9/88 (10.2)
Hydrocephalus	8/88 (9.0)
**EEG (DONE IN 95 PATIENTS)**	
Typical hypsarryhthmia	31/95 (32.6)
Generalized or multifocal spikes	38 (40.0)
Focal spikes	14 (14.7)
Burst suppression	6 (6.3)

**Figure 1:**
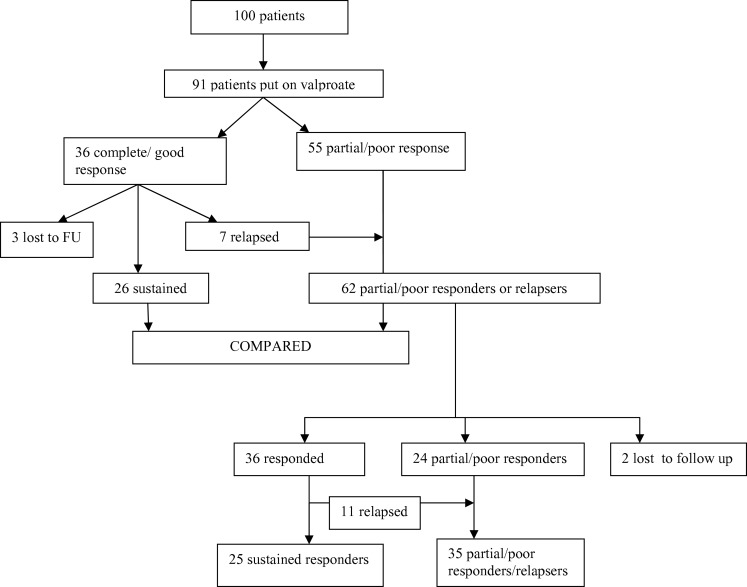
**Showing enrollment of patients and outcomes**.

Of the 100 enrolled patients, nine were already on treatment, so could not be given treatment according to the study protocol. Thus, a total of 91 patients were put on valproate, while awaiting investigations. Of these, 36 (39.5%) showed initial good response (response categories 4 or 5), while 55 patients showed partial or poor response. However of the 36, seven patients relapsed within 3 months on same dose of valproate, three were lost to follow up, and only 26 (29.5%) patients continued to have a sustained good response (Figure 1). Patients were followed up for at least 6 months. No significant adverse effects were noted and routine monitoring of liver functions and valproate drug level was not done. The reason for loss to follow up could not be ascertained as these patients could not be contacted.

Clinical, demographic, and risk factors were compared in those showing good (26 patients) and partial/poor response/relapse (62 patients) by univariate analysis to look for factors associated with good sustained response to valproate (Table 2). Comparison of means was done by ANOVA and comparison of proportions by the Chi-Square test. Older age at onset of spasms (*p* = 0.021) and typical hypsarrhythmia on EEG (*p* = 0.001) were found to be significantly associated with a sustained good response to valproate therapy, while history of delayed cry at birth was associated with a poor response (*p* = 0.040).

**Table 2:** **Comparison of patients who showed a sustained response to valproate with those who had partial/poor response or relapse**.

Factors	Partial/poor response *n* = 62 (%)	Good response *n* = 26 (%)	Odds ratio (95% Confidence limits)	*p* Value
Mean age at onset (in months; SD)^1^	4.49 (4.46)	8.17 (10.44)	–	0.021*
Mean age at presentation (in months) mean (SD)^1^	13.84 (9.18)	17.69 (15.29)	–	0.149
Mean delay in presentation (in months) mean (SD)^1^	9.40 (8.68)	9.78 (10.15)	–	0.858
Male sex^2^	55 (78.6)	17 (68.0)	0.55 (0.20–1.50)	0.181
Urban residence^2^	28 (45.1)	13 (50.0)	1.2 (0.4–3.3)	0.678
Hospital birth^2^	35 (56.4)	13 (50.0)	0.77 (0.3–2.1)	0.579
Adverse perinatal/neonatal events^2^	45 (72.5)	19 (73.0)	1.03 (0.3–3.3)	0.961
Preterm gestation^2^	5 (8.0)	2 (7.6)	0.95 (0.1–6.1)	1.000
LBW^2^	17 (27.4)	4 (15.3)	0.48 (0.1–1.8)	0.226
Delayed cry at birth^2^	43 (69.3)	12 (46.1)	0.38 (0.1–1.1)	0.040*
Neonatal illness^2^	30 (48.3)	16 (61.5)	1.71 (0.6–4.8)	0.259
Cryptogenic etiology^2^	4 (6.4)	5 (19.2)	3.45 (0.7–17.3)	0.117
Adverse post-neonatal events^2^	9 (14.5)	4 (15.3)	1.07 (0.2–4.4)	1.000
“Typical” infantile spasms^2^	42 (67.7)	19 (73.0)	1.3 (0.4–4.0)	0.620
Spasms in series^2^	33 (53.2)	14 (53.8)	1.03 (0.4–2.8)	0.957
Spasms mostly on awakening^2^	53 (85.4)	20 (76.9)	0.57 (0.2–2.1)	0.360
Normal motor development^2^	16 (25.8)	5 (19.2)	0.68 (0.2–2.3)	0.509
Normal mental development^2^	20 (32.2)	9 (34.6)	1.11 (0.4–3.2)	0.830
Normal social development^2^	20 (32.2)	11 (42.3)	1.54 (0.5–4.4)	0.367
**Associated features**				
Developmental delay at onset of spasms^2^	48 (77.4)	20 (76.9)	0.97 (0.3–3.3)	0.959
Any dysmorphic feature^2^	17 (27.4)	4 (15.3)	0.5 (0.1–1.8)	0.255
Microcephaly^2^	32 (51.6)	12 (46.1)	0.8 (0.3–2.2)	0.640
Abnormal motor examination^2^	45 (72.5)	18 (69.2)	0.85 (0.3–2.6)	0.750
Other seizures at presentation^2^	14 (22.5)	6 (23.0)	1.03 (0.3–3.4)	0.959
Typical hypsarrhythmia on EEG (85/95)^2^	14/60 (23.3)	15/25 (60.0)	4.93 (1.6–15.2)	0.001*
Neuroimaging suggestive of HIE (*n* = 82/88)^2^	24/59 (40.6)	6/23 (26.0)	0.51 (0.15–1.66)	0.217

*^1^Comparison of means by ANOVA; figures indicate mean (SD)*.

*^2^Comparison of proportions by chi-square test; figures indicate number of patients (%)*.

*Significant.

Patients who showed an initial partial/poor response to valproate, or later relapsed after initial good response were then put on hormonal therapy in addition. A total of 62 children were put on hormonal therapy, of which 36 (58.06%) showed good initial response but of these 11 later relapsed after hormonal therapy was discontinued and two patients were lost to follow up. So only 23/62 (37.1%) could be counted as having a sustained response to hormonal treatment (Figure 1). Children who showed partial/poor response to hormonal therapy or those who relapsed were tried on other antiepileptic drugs such as vigabatrin and lamotrigine.

## Discussion

The present study comprised of a relatively large number of patients compared with some previous studies; 47 children by Matsuo et al., 2001; Sharma and Vishwanthan, 2008), 26 children by Sharma and Vishwanthan, 2008; Goldstein and Slomski, 2008), 28 children by Goldstein and Slomski, 2008; Cohen-Sadan et al., 2009). Criteria for definition of West syndrome were similar to previous studies and hypsarrhythmia on EEG is not considered essential for the diagnosis (Lux and Osborne, 2004). An in depth study of all possible factors – demographic, clinical, and investigative, which can affect response to therapy was made. However, our outcome was only seizure control rather than reversal of EEG features and developmental outcome as these investigations could not be repeated in the majority of our patients. Seizure control has been shown to correlate with improvement both of EEG and development. Another limitation was the way study factors were recorded. Since medical records are usually not available the family’s word was taken at face value about “delayed cry,” low birth weight etc. Further, this is an unblinded, uncontrolled, and non-randomized study.

The mean age of onset was similar to that found in other studies. It was 5.77 months on an average compared with 5.5 months in a previous study from Israel (Singhi and Ray, 2005) and 6.1 ± 3.4 months in an Indian study (Kalra and Passi, 1998). The delay in presentation observed by us was also observed by other Indian workers. We find that parents often do not realize that the “startling” episodes are seizures. The pediatricians first seeing these children have to specifically ask if the child “startles” without any stimulus. Demographic and socioeconomic characteristics may merely represent the profile of patients seen in our hospital. Perinatal insults formed the major etiologic group compared with antenatal and postnatal timing of insult. This has been the conclusion in many of the studies done previously (Kalra and Passi, 1998; MacKay et al., 2004; Tsuji et al., 2007).

The type of spasms were flexor in the majority. This was noted by previous workers also. About 4/5th of patients had spasms especially on awakening. However only slightly more than half gave a typical history of spasms occurring in series. Such observations have not been remarked on in earlier studies. Lux and Osborne (2004) categorized patients with spasms occurring singly rather than in clusters as a separate group – infantile spasms single spasm variant (ISSV). Associated developmental delay was present in almost 90% of our patients while the rest had developmental regression after onset of spasms. Other types of seizures at presentation were present in 20% – very similar to the 31/165 (18.7%) seen by Singhi and Ray (Kalra and Passi, 1998).

Encephalomalacia/sequelae to perinatal hypoxic ischemic encephalopathy was the major finding on neuroimaging seen in 32.4% cases, the 2nd commonest finding being just atrophy. Typical hypsarrhythmia was seen in a relatively small number of patients (31.5%) as also seen by other Indian workers (Kalra and Passi, 1998; Kalra et al., 2002; Tsuji et al., 2007). They however qualified as West syndrome since developmental delay was present in almost all the patients.

Availability of newer antiepileptic drugs has led to various treatment regimens for West syndrome being tried all over the world, topiramate (Peltzer et al., 2009), zonisamide (Yum and Ko, 2009), pyridoxine (Auvichayapat et al., 2007), benzodiazepines (Sharma, 2012), lamotrigine (Gumus et al., 2007), and levetiracetam (Veggiotti et al., 1994). Some workers have tried high dose valproate in West syndrome (Seimes et al., 1988; Pratz et al., 1991; Ohtsuka et al., 1992; Sharma, 2012) in. However, the number of patients treated in these studies was small. Seimes et al. (1988) using progressively higher doses of valproate could control spasms in 16 of 22 patients over a period of 6 months. We did not go on to high dose valproate because we did not want to further delay starting the “standard” treatment (hormonal). It is possible that higher dosing may have produced a response in higher proportion of patients.

We tried valproate initially in our patients only while they were undergoing investigations. Patients were recalled every 2 weeks and those who showed partial or poor response to valproate were immediately shifted to the next line of therapy. An 80% response in seizures was taken as meaningful because we feel that with a lesser response, the physician would be prompted to use another drug. Previous workers have taken a 50% or greater response in seizures as a good response (Kalra et al., 2002).

Almost 2/5th of our patients had a good response to valproate initially within 2 weeks but sustained response to valproate was seen in less than 30%. Later age of onset was found to be associated with good sustained response to treatment. This was found by Riikonen also (Lux et al., 2005). Hypsarrhythmia is usually found early in the course of West syndrome, being later superceded by other abnormalities. Atypical or asymmetric hypsarrhythmia therefore may be associated with poor seizure control (Kalra et al., 2002). A history of delayed cry often indicates perinatal asphyxia and was the most common type of “symptomatic” West syndrome, which carries a worse prognosis.

Efficacy of valproate has not been adequately studied in West syndrome and no randomized controlled trial data is available (Riikonen, 2010) although some earlier workers have found it to be useful in high doses. In this relatively large study, we found a favorable response in a significant proportion of patients even with usual doses of valproate used alone. It must be borne in mind that there may be completely different disease cohorts within our patients with West syndrome, some of whom may respond to valproate. We have tried to delineate this subset of patients through this study. Proportion of initial responders who relapsed were higher with hormonal therapy than with valproate (30.5 vs. 19.4%). Hence, with such a significant response to a relatively less toxic drug, we feel that there is a case for further elucidating the role of valproate in initial management of selected patients with West syndrome through an appropriately powered randomized controlled trial possibly in different dose ranges.

## Conflict of Interest Statement

The authors declare that the research was conducted in the absence of any commercial or financial relationships that could be construed as a potential conflict of interest.
